# *In Vivo* Evaluation of Blood Based and Reference Tissue Based PET Quantifications of [^11^C]DASB in the Canine Brain

**DOI:** 10.1371/journal.pone.0148943

**Published:** 2016-02-09

**Authors:** Nick Van Laeken, Olivia Taylor, Ingeborgh Polis, Sara Neyt, Ken Kersemans, Andre Dobbeleir, Jimmy Saunders, Ingeborg Goethals, Kathelijne Peremans, Filip De Vos

**Affiliations:** 1 Department of Radiopharmacy, Ghent University, Ghent, Belgium; 2 Department of Medical Imaging and Small Animal Orthopedics, Ghent University, Ghent, Belgium; 3 Department of Medicine and Clinical Biology of Small Animals, Ghent University, Ghent, Belgium; 4 Department of Radiology and Nuclear Medicine, Ghent University Hospital, Ghent, Belgium; Banner Alzheimer's Institute, UNITED STATES

## Abstract

This first-in-dog study evaluates the use of the PET-radioligand [^11^C]DASB to image the density and availability of the serotonin transporter (SERT) in the canine brain. Imaging the serotonergic system could improve diagnosis and therapy of multiple canine behavioural disorders. Furthermore, as many similarities are reported between several human neuropsychiatric conditions and naturally occurring canine behavioural disorders, making this tracer available for use in dogs also provide researchers an interesting non-primate animal model to investigate human disorders. Five adult beagles underwent a 90 minutes dynamic PET scan and arterial whole blood was sampled throughout the scan. For each ROI, the distribution volume (V_T_), obtained via the one- and two- tissue compartment model (1-TC, 2-TC) and the Logan Plot, was calculated and the goodness-of-fit was evaluated by the Akaike Information Criterion (AIC). For the preferred compartmental model BP_ND_ values were estimated and compared with those derived by four reference tissue models: 4-parameter RTM, SRTM2, MRTM2 and the Logan reference tissue model. The 2-TC model indicated in 61% of the ROIs a better fit compared to the 1-TC model. The Logan plot produced almost identical V_T_ values and can be used as an alternative. Compared with the 2-TC model, all investigated reference tissue models showed high correlations but small underestimations of the BP_ND_-parameter. The highest correlation was achieved with the Logan reference tissue model (Y = 0.9266 x + 0.0257; R^2^ = 0.9722). Therefore, this model can be put forward as a non-invasive standard model for future PET-experiments with [^11^C]DASB in dogs.

## Introduction

The serotonin transporter (SERT) is a plasma membrane transporter that belongs to the neurotransmitter sodium symporter (NSS) family. By using the energy stored in the transmembrane ion gradients of Na^+^, Cl^-^ and K^+^, it selectively transports extracellular serotonin from the synaptic cleft back into the presynaptic nerve terminals. Thereby SERT terminates the neurotransmission at extracellular receptor sites and makes it available for recycling into new synaptic vesicles [1–3].

Alterations in brain SERT density and availability are reported to be involved in both the pathophysiology and treatment of a variety of pathological and neurological disorders such as major depressive disorder[4], obsessive compulsive disorder[5], social phobia [6,7], Parkinson’s disease [8] and Alzheimer’s disease [9]. Intense research during the past decades to image this transporter with positron emission tomography (PET) points out that the radiotracer [^11^C]-3-amino-4-(2-dimethylaminomethyl-phenylsulfanyl)-benzonitrile or [^11^C]DASB, introduced in 2000 by Wilson and co-workers [10], can be considered as an appropriate candidate. This tracer combines several major prerequisites, such as a high affinity (Ki 1.1 nM), an excellent selectivity (NET/SERT 1230—DAT/SERT 1300), a high specific to nonspecific binding ratio, a reversible high brain uptake and a binding equilibrium within a reasonable time frame. [10,11]

Up to now, [^11^C]DASB has been extensively used in rodents [12–14], pigs [15], cats [16], non-human primates [13,17,18], and humans [19–21], but despite the potential added value, this tracer has never been used in dogs. Imaging the serotonin transporter, or the serotonergic system in general, could improve diagnosis and therapy of canine behavioural disorders that may have an impact on the daily life of the human society. Furthermore, naturally occurring canine behavioural and human neuropsychiatric disorders, such as anxiety [22], aggressive [23] and compulsive [24,25] disorders, share many similarities which ensures that dogs represent a more practical and available alternative to other laboratory animals such as rodents or nonhuman primates.

The aim of this study is to present an evaluation of the [^11^C]DASB PET data obtained in beagles, where different standard kinetic models for quantification were assessed using a metabolite corrected arterial plasma input function. Furthermore, to evaluate whether or not invasive blood sampling can be excluded in the future, the validity of several reference tissue models was also investigated.

## Materials and Methods

### Experimental animals

The study was approved by the Ethical Committee of Ghent University (EC approval 2013/133). Five (four male, one female) healthy adult laboratory beagles (age 6 ± 2 years, weight 20 ± 10 kg, Marshall farms) were included in this study. All scans were performed between October 10, 2013 and January 30, 2014. Dogs were housed as pairs in kennels of 2.6 m^2^ and had unlimited access to water. The dogs were fasted for at least 12 hours before the PET/CT scan. They were sedated with an i.m. injection of dexmedetomidine (375 μg/m^2^ body surface area, Dexdomitor®, Orion Corporation, Espoo, Finland), transported to the PET-center of the Ghent University hospital and placed on the bed of the PET/CT scanner (sternal recumbency with the front limbs extended caudally). To induce general anesthesia with propofol (2–3 mg/kg, depending on response, Propovet®, Abbott Laboratories, Queenborough, UK), a 22G venous catheter was placed in one of the cephalic veins. Thereafter, anesthesia was maintained with a mixture of 1.2–1.4% isolurane (Isoflo®, Abbott Laboratories) in oxygen using a rebreathing system. A 22G arterial catheter was placed in one of the arteries dorsalis pedis to perform arterial blood sampling. Continuous monitoring of body temperature and cardiorespiratory functions by pulse oximetry and capnography was performed during and after anesthesia until the animals were fully awake. After completion of the study protocol the beagles were kept alive and made available for future research.

### Radiosynthesis

The serotonin transporter ligand [^11^C]DASB was synthesized by N-methylation of the precursor N-desmethyl-DASB (50 μg, ABX, Radeberg, Germany) with [^11^C]methyl triflate using established methods. [26] This resulted in moderate to high activities of 2405 ± 1406 MBq and high radiochemical purities of more than 99%. The specific radioactivities measured with analytical HPLC were 74 ± 50 GBq/μmol at the end of synthesis and 43 ± 34 GBq/μmol at the time of tracer injection. As all beagles were injected with a dose of 289 ± 60 MBq, the SERT occupancy, calculated via the method of Hume and colleagues (1998) and using the mean ED_50_ value of 56 nmol/kg, was 1.09 ± 0.88% [12,27].

### [^11^C]DASB PET/CT scanning protocol

All dogs were scanned with a Gemini PET/CT imaging system (Philips Co., Eindhoven, The Netherlands), which consists of a gadolinium oxyorthosilicate full-ring PET scanner with 5 mm in-plane spatial resolution. After conducting a low dose CT survey (16-slice helical scan, 120 kV, 30 mA, FOV 600 mm, 0.5 s rotation time, pitch of 0.9, collimation 16 x 1.5 mm) for attenuation correction, dynamic emission recordings in list mode were initiated on bolus injection of 289 ± 60 MBq [^11^C]DASB. Emission data were reconstructed as 34 successive frames of increasing duration (6 x 10, 8 x 30, 5 x 120, 15 x 300 s) using the iterative 3D-RAMLA (Row Action Maximum Likelihood Algorithm) algorithm provided by Philips and the resulting voxel size of the images was 1x1x1 mm^3^. During the 90 minutes PET scan, arterial whole blood samples (1–2 mL) were taken manually into heparinized syringes at several time points with increasing intervals (15, 30 and 45 seconds, 1.25, 1.5, 1.75, 2, 2.5, 5, 10, 12 and 20 minutes, and every ten minutes thereafter) and collected in K_3_EDTA tubes. After centrifugation of the blood samples (5 min, 5200 rpm), the plasma fraction was separated from the blood cells and the plasma activity was measured using a calibrated 3x3 inch NaI(Tl) scintillation detector (Canberra, Meriden, Connecticut, USA). For each dog, the parent compound fraction was measured at 4–9 time points using a validated solid phase extraction (SPE) procedure.[19] OASIS HLB Plus cartridges (225 mg, 60 μm, Waters Corporation, Milford, MA, U.S.A) were prewashed with consecutively 5 mL of each tetrahydrofuran (THF), ethanol, and water. After applying the plasma sample on the cartridge, the cartridge was washed with successively 5 mL 5% methanol (MeOH) in water, 5 mL 22% acetonitrile (CH_3_CN) in water containing 0.1 N ammonium formate, and 5 mL THF. The ratio of the activity in the THF fraction to the total activity was determined and equals the fraction of plasma radioactivity representing unchanged [^11^C]DASB.

### Regions of interest

Prior to the day of the PET-scan, each dog underwent a series of 3D high resolution T1-weighted anatomical images (3D MPRAGE sequence, 176 sagital slices, TR = 2250 ms, TE = 4.18 ms, TI = 900 ms, parallel acquisition method = GRAPPA with acceleration factor = 2, matrix size = 256 x 256, FOV = 220 mm, flip angle = 8°, voxel size = 1 x 1 x 1 mm^3^), obtained on a 3T Magnetom Trio Tim System MRI scanner (Siemens Medical Systems, Erlangen, Germany) using a phased-array spine coil and a phased-array body matrix coil. In order to provide anatomical information, these images were subsequently coregistered with the PET image using the PMOD software version 3.0 (PMOD Technologies Ltd., Zurich, Switzerland). Based on two dog brain atlases [28,29], 18 regions of interest (ROIs) were manually delineated on dorsal planes: anterior cingulate gyrus, basal ganglia left, basal ganglia right, cerebellar cortex (vermis excluded), frontal cortex left, frontal cortex right, hippocampus left, hippocampus right, occipital cortex left, occipital cortex right, parietal cortex left, parietal cortex right, posterior cingulate gyrus, brainstem region containing the raphe nuclei, temporal cortex left, temporal cortex right, thalamus left and thalamus right. Within the cortex only grey matter was included. For each ROI a time-activity curve was calculated in PMOD by determining the radioactivity concentration for each frame, correcting it for decay, and plotting it versus time.

### PET-data quantification

All kinetic modeling was performed with PMOD’s Kinetic Tool (version 3.405) and comparisons between the models were done according to a commonly used methodology.[30] The volume of distribution (V_T_), representing the ratio of the concentration of radiotracer in a particular ROI to the concentration in plasma at equilibrium, and related standard error coefficients of variation (COV), were calculated for each ROI based on two standard full kinetic compartmental models, the single- and two-tissue compartment (1-TC and 2-TC) model [31,32], and a graphical analysis technique, the Logan plot [33]. The derivations, at equilibrium, of the relationship between the rate constants and V_T_ can be calculated as follows [34]:

1-TC model:

$$V_{T}=\frac{K_{1}}{k_{2}}$$ (1)

2-TC model:

$$V_{T}=\frac{K_{1}}{k_{2}}\;(1+\frac{k_{3}}{k_{4}})$$ (2)

For both standard compartmental models the brain activity was corrected for the contribution of plasma activity assuming a cerebral blood volume in the regions of interest fixed at 0.05 mL/cm^3^ [35]. The goodness-of-fit was evaluated using the Akaike Information Criterion (AIC) [36], whereby lower AIC values indicate a better fit and a penalty was given for increasing the number of parameters in the model.

In order to validate the use of a reference tissue model for future PET-experiments with [^11^C]DASB, binding potentials were estimated for the preferred compartmental model and several reference tissue models. The binding potential (BP_ND_) refers to the ratio at equilibrium of specifically bound radioligand to that of nondisplaceable radioligand in tissue [34] and therefore requires the presence of a region devoid of receptors (i.e., reference region). For PET studies with [^11^C]DASB, the cerebellum has been put forward over the years as the reference region of choice, but still contains a considerable displaceable fraction. Notwithstanding this, an autoradiography study with [^3^H]cyanoimipramine, stated that the specific SERT binding is much higher in the cerebellar vermis (8.4 fmol/mg) compared with the cerebellar gray matter (1.25 fmol/mg). [37] Therefore we adapted the recommendation of Meyer [38] to include the posterior half of the cerebellar cortex in the delineation of the ROI, thereby excluding the vermis and keeping distance from white matter, venous sinuses and the occipital cortex. For the preferred compartmental model BP_ND_ can be calculated as:

$${BP}_{ND}=\frac{(V_{T}-V_{ND)}}{V_{ND}}=\frac{V_{T}}{V_{ref}}-1$$ (3)

Four reference tissue models were included in the study: the 4-parameter reference tissue model (RTM)[39], the two-steps simplified reference tissue model (SRTM2)[40,41], the 2-parameter multilinear reference tissue model (MRTM2)[42] and the Logan reference tissue model [43]. For all these models, the degree to which they could reproduce the BP_ND_ values observed using the 2-TC model was investigated. Thereby, the BP_ND_ and COV was calculated for each ROI using a fixed k_2_’ value representing the tissue clearance rate from the reference region. For MRTM2, MRTM [31,42] was used to calculate k_2_’ and for SRTM2 and the Logan reference tissue model, SRTM [31,40] was used to calculate k_2_’. In both cases, a fixed k_2_’ was determined as the mean value of five high binding regions: raphe nuclei, thalamus left, thalamus right, basal ganglia left and basal ganglia right. For comparison between the reference tissue models and the preferred compartmental model only fits with a COV smaller than 25% were taken into account.

### Statistical analysis

Statistical analysis was performed using IBM SPSS Statistics version 22 (New York, US), whereby results are considered statistically significant if the p-value is under .05.

## Results

### Plasma analysis

**Fig 1:**
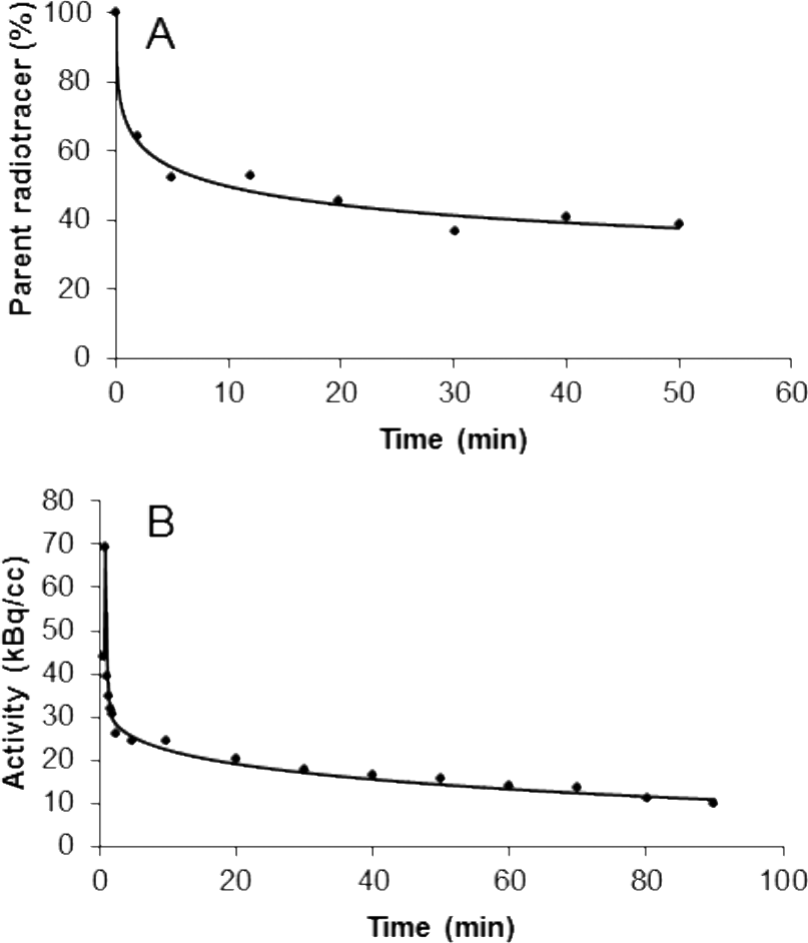
**(A) Time course for the percentage of radioactivity in plasma (mean ± SD) corresponding to unchanged [^11^C]DASB–(B) A representative metabolite corrected plasma inputfunction**.

During the first minutes after tracer injection, the fraction of unmetabolized [^11^C]DASB in arterial plasma rapidly declined to 53 ± 3% at 5 minutes. Thereafter the rate of metabolism continuously decreased (Fig 1A). Because of less accurate count statistics at later time points in three of the dogs, mean values at each time-point were used to set up the curve. A Hill-type function (Eq 4) could be fitted to the fraction of parent radiotracer (f_parent_) in order to subsequently enable estimation of the metabolite corrected plasma input functions.

$$f_{parent}\left(t\right)=1-\left(\frac{0.93\;t^{0.34}}{t^{0.34}+7.51}\right)\;\text{with t representing time},\;\text{expressed in seconds}$$ (4)

The tail of these plasma input functions could be, in turn, fitted individually using a bi-exponential function, which resulted in excellent fits (Fig 1B). Arterial plasma activity concentration reached a peak within the first minute and was then followed by a rapid distribution phase. Subsequently, the rate of decrease of radioactivity in plasma significantly diminished towards a half-life of over 45 minutes in every laboratory beagle.

### Brain analysis

**Fig 2:**
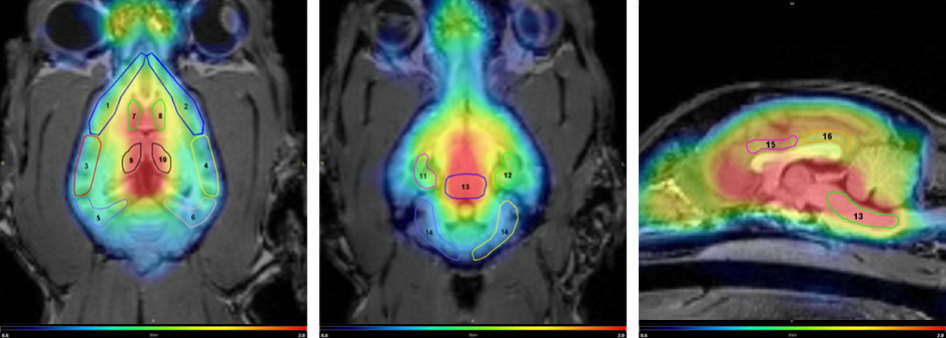
Distribution of [11C]DASB in the canine brain: dorsal and sagital sections of a summed PET-image, fused with MRI. Regions of interest delineated on the image: 1. Frontal cortex right – 2. Frontal cortex left – 3. Temporal cortex right – 4. Temporal cortex left – 5. Occipital cortex right – 6. Occipital cortex left – 7. Basal ganglia right – 8. Basal ganglia left – 9. Thalamus right – 10. Thalamus left – 11. Hippocampus right – 12. Hippocampus left – 13. Region in brainstem containing raphe nuclei – 14. Cerebellar cortex, vermis excluded – 15. Anterior cingulate gyrus – 16. Posterior cingulate gyrus.

**Fig 3:**
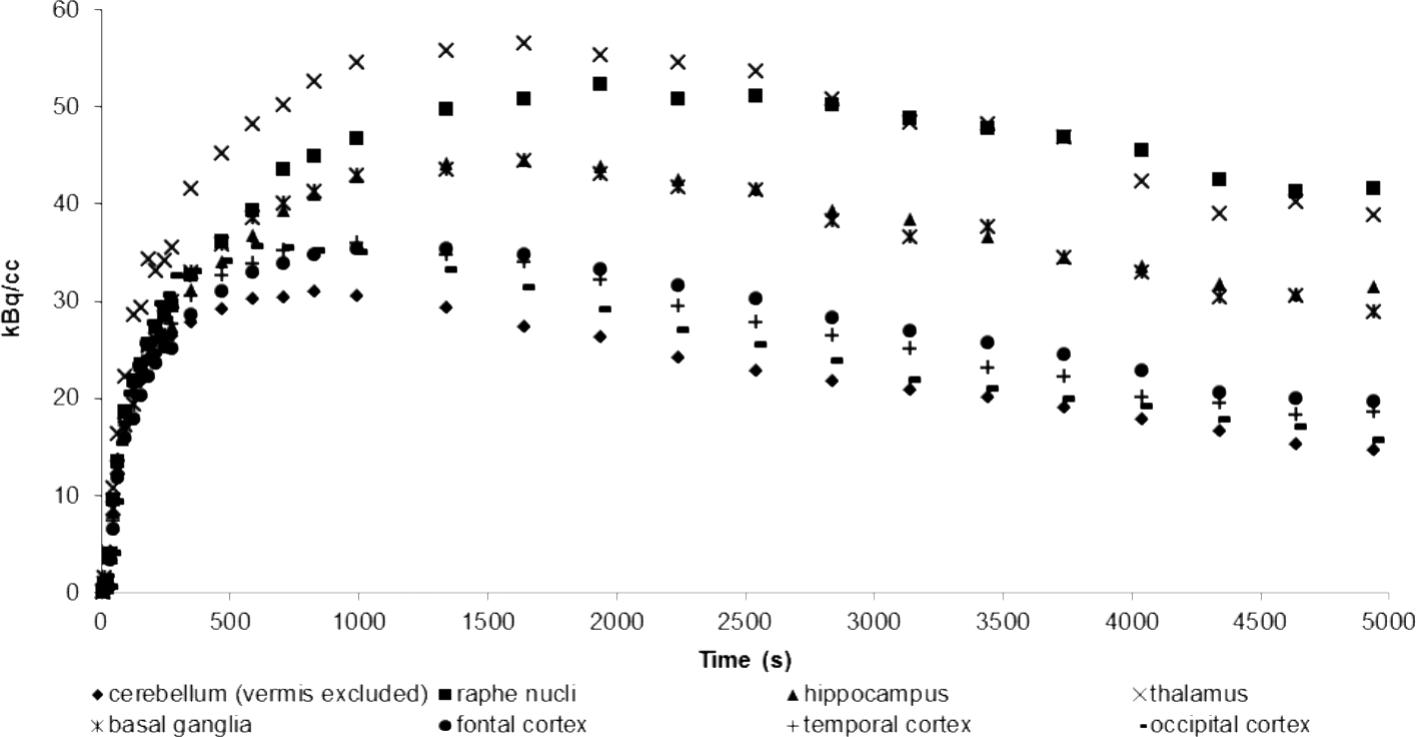
Regional time-activity curves measured after injection of 29 MBq/kg [11C]DASB in a six year old female beagle.

Dynamic [^11^C]DASB PET images revealed an *in vivo* distribution consistent with the known distribution of SERT sites in other species, thereby observing high radioactivity levels in the raphe nuclei and thalamus, intermediate levels in the hippocampus and basal ganglia and lower levels in the cortical regions and the anterior and posterior cingulate gyri (Figs 2 and 3). Provided that the vermis was excluded, lowest radioactivity levels were observed in the cerebellar cortex.

### Kinetic analysis

**Fig 4:**
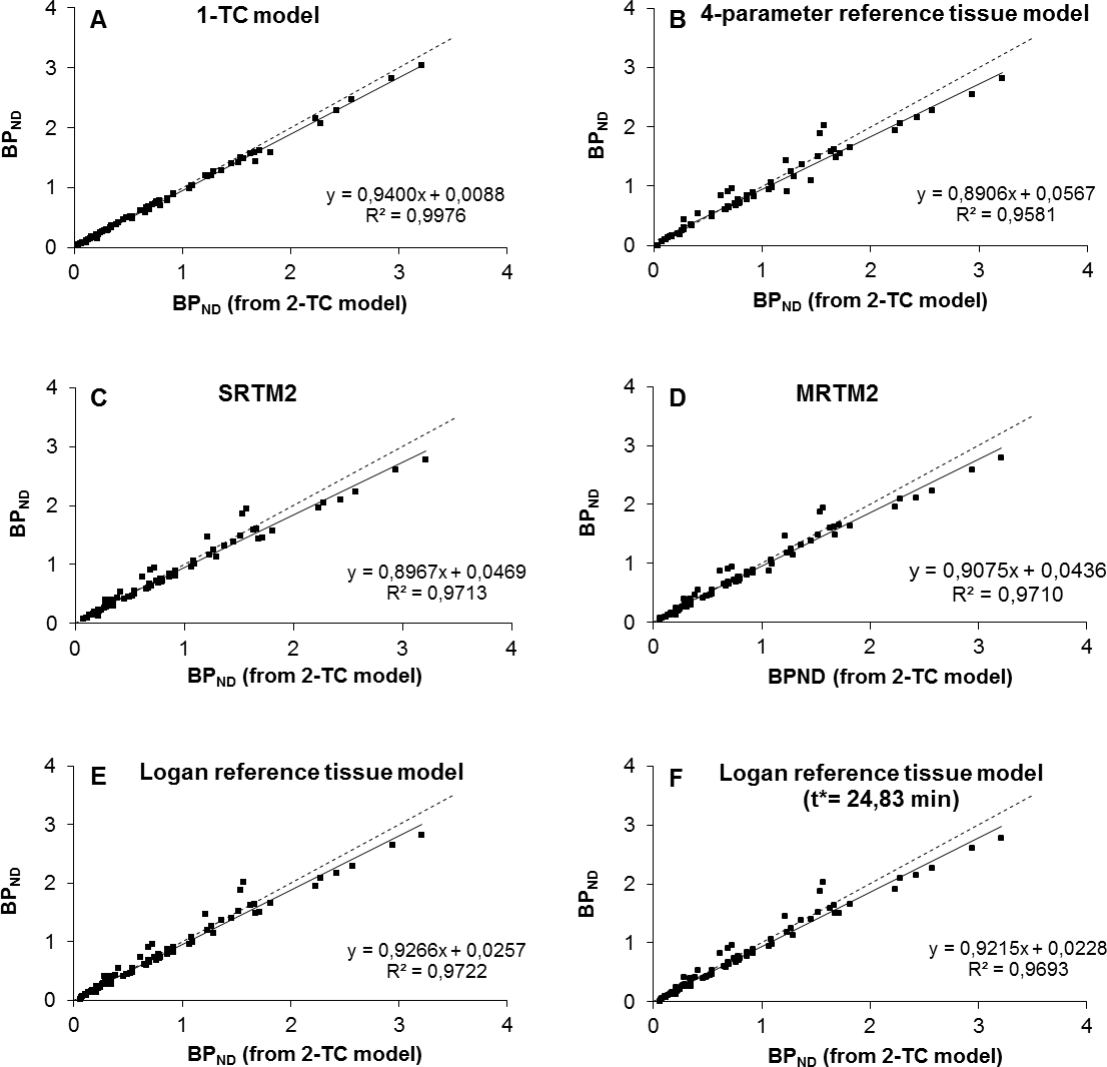
Correlations between [11C]DASB binding potentials (BPND) calculated from PET data using different kinetic models.

For all ROIs observed, V_T_ values (mean ± SD), obtained with the 1-TC and 2-TC model, gave maximum COV of respectively 4.40% and 6.24% and were significantly different from each other (paired sample t-test, two-tailed p-value = 0.00004). The 2-TC model showed superior fitting compared to the 1-TC as judged by lower AIC values in 61% of the observed brain regions (Table 1). Especially for the regions with high (raphe nuclei and thalamus) and intermediate (hippocampus and basal ganglia) SERT densities, the 2-TC model was preferred in 89% of the cases. Plotting BP_ND_ values obtained with 1-TC versus these obtained with 2-TC (Fig 4A), indicates small underestimations with the 1-TC model. However, the results are highly correlated with a Pearson product moment correlation coefficient (R^2^) of 0.9976.

**Table 1:** Distribution Volumes (Mean ± SD) and AIC values (Mean) for Single- and Two-Tissue Compartmental Models and the Logan Plot.

	1-TC	AIC	2-TC	AIC	Logan	Logan (t* = 24,83 min)	Ratio 1-TC/2-TC	Ratio Logan/2-TC	Ratio Logan (t* = 24,83 min)/2-TC
**Raphe nuclei**	3.41±0.31	49	3.63±0.46	-22	3.53±0.41	3.54±0.41	0.94	0.97	0.97
**Hippocampus L**	2.37±0.40	52	2.48±0.48	10	2.44±0.46	2.44±0.47	0.95	0.98	0.98
**Hippocampus R**	2.28±0.18	42	2.38±0.21	2	2.37±0.20	2.37±0.22	0.96	1.00	1.00
**Thalamus L**	3.42±0.65	53	3.63±0.76	10	3.49±0.67	3.49±0.69	0.94	0.96	0.96
**Thalamus R**	3.34±0.33	48	3.53±0.42	2	3.44±0.38	3.44±0.38	0.95	0.98	0.97
**Basal ganglia L**	2.81±0.82	41	2.94±0.95	6	2.87±0.85	2.88±0.87	0.96	0.97	0.98
**Basal ganglia R**	2.69±0.52	39	2.82±0.61	-2	2.76±0.54	2.76±0.55	0.96	0.98	0.98
**ACG**	1.78±0.22	56	1.83±0.26	34	1.80±0.28	1.80±0.29	0.97	0.98	0.98
**PCG**	1.58±0.11	54	1.62±0.14	38	1.61±0.19	1.60±0.20	0.97	0.99	0.99
**Frontal cortex L**	1.87±0.17	43	1.93±0.24	14	1.93±0.26	1.93±0.27	0.96	1.00	1.00
**Frontal cortex R**	1.88±0.17	36	1.94±0.23	5	1.95±0.24	1.95±0.25	0.97	1.01	1.00
**Temporal cortex L**	1.64±0.15	48	1.68±0.17	32	1.67±0.19	1.67±0.20	0.98	0.99	0.99
**Temporal cortex R**	1.62±0.05	45	1.65±0.07	28	1.64±0.09	1.64±0.10	0.98	1.00	0.99
**Occipital cortex L**	1.36±0.12	49	1.41±0.16	37	1.37±0.18	1.37±0.18	0.97	0.97	0.97
**Occipital cortex R**	1.36±0.11	53	1.39±0.10	39	1.37±0.16	1.36±0.17	0.98	0.98	0.98
**Parietal cortex L**	1.54±0.19	46	1.57±0.20	29	1.55±0.17	1.54±0.17	0.98	0.98	0.98
**Parietal cortex R**	1.49±0.15	44	1.52±0.18	26	1.51±0.20	1.51±0.21	0.98	0.99	0.99
**Cerebellar cortex (vermis excluded)**	1.27±0.10	37	1.29±0.07	27	1.31±0.10	1.31±0.10	0.98	1.02	1.01
						**Mean ratio**	**0.97**	**0.99**	**0.99**

L = left, R = right, ACG = anterior cingulate gyrus, PCG = posterior cingulate gyrus

Maximum COV for the V_T_ values calculated with the Logan plot was 1.98% and no relevant under- or overestimations were observed in any of the ROIs. The results demonstrated a strong correlation with those obtained via the 2-TC model, which was represented by the mean ratio between estimates outputs of 0.99. Fixing the starting point for linearization to the highest value observed across all ROIs, 24.83 min, had no significant effect on the outcomes (paired t-test, two-tailed p-value = 0.668).

In order to investigate the degree to which a reference tissue model could reproduce the observed BP_ND_ values using the 2-TC model, BP_ND_ values were calculated with the 4-parameter RTM, SRTM2, MRTM2, and the Logan reference tissue model (start time for the linearization as a free variable or fixed at 24.83 min), thereby using the cerebellar cortex (vermis excluded) as a reference region. Fitted values with a COV < 25% were plotted against the corresponding ones obtained with the preferred 2-TC model (Fig 4B–4F). The 4 parameter reference tissue model reached convergence in 80 of 85 regions, although it was the model with the highest sensitivity to noisy data as 21 of the remaining regions show COV > 25%. The SRTM2 and MRTM2 model both failed to reach convergence in only 4 regions and had acceptable fits (COV < 25%) in respectively 77 and 78 regions. As three fits with MRTM failed to have COV < 25% for calculation of k2’ values, these regions were excluded in the calculation of the fixed k2’ value to use with MRTM2. For the Logan reference tissue model convergence was reached in 80 regions and all had acceptable fits, independently from the start time for linearization fixed to 24.83 min or not.

Compared with the 2-TC model, all investigated reference tissue models show small, but significant (One way repeated measures ANOVA, Pairwise comparisons with Bonferroni correction, p-values ≤ 0.026) underestimations of BP_ND_, ranging from 4.1% to 5.6%. However all data were highly correlated, stated by R² values ranging from 0.9581 (4-parameter RTM) to 0.9722 (Logan). The best correlation was achieved with the Logan reference tissue model, leaving the time from which the regression was computed as a free variable (Y = 0.9266 x + 0.0257; R² = 0.9722).

## Discussion

To our best knowledge, this is the first in-dog study that investigates the kinetic properties of the radiotracer [^11^C]DASB in the canine brain and validates the use of several reference tissue models as a noninvasive alternative to standard compartmental modeling.

After tracer injection, [^11^C]DASB revealed a regional distribution pattern in the canine brain which was consistent with the one observed in humans, non-human primates, cats and rodents. As also observed in humans [44], radioactivity peaked earlier in the low binding regions (10–20 min) than in the high binding regions (27–37 min). Despite the previously reported high brain uptake of [^11^C]DASB in the hypothalamus of several species, limited spatial resolution of PET and partial volume effects prevented us from including this region in the study. The olfactory bulb was also not included in the study due to the complex structure of blood vessels in this region causing problems with the delineation of this ROI.

According to the Akaike Information Criterion the 2-TC model indicated in 61% of the ROIs a better fit compared to the 1-TC model, especially for regions with high and intermediate SERT-densities such as the raphe, the thalamus, the hippocampus and the basal ganglia. Given the nearly identical V_T_ values derived by the Logan plot analysis, this graphical analysis model can be used as an alternative to the 2-TC model.

Compared to laboratory beagles, PET studies with [^11^C]DASB in humans [19] or rhesus monkeys [13] often report a high degree of nonconvergence when an unconstrained 4 parameter 2-TC model is used and, in these cases, the 1-TC model is put forward as the preferred full kinetic compartmental model. Despite this, our results are consistent with two published studies with [^11^C]DASB in healthy volunteers where, in the one study[45], it was observed that, based on the AIC, a constrained 2-TC model indicated a better fit in 58% of the examined ROIs, and in the other study[44] that the 1-TC model did not adequately describe the rising portion of the time-activity curve. Comparisons with the kinetic analysis approach in other species [13–17] is more complicated due to a lack of studies that investigated and justified the use of a specific full kinetic compartmental model or reference tissue model in those particular species.

## Conclusion

We found that in the canine brain the [^11^C]DASB radiotracer follows two-tissue compartment kinetics. For future experiments invasive arterial blood sampling can be avoided by using one of the investigated reference tissue models (highest correlation observed with Logan reference tissue model), however small underestimations of the BP_ND_ parameter must be taken into account.

## Supporting Information

S1 File Revalidation of the SPE purification procedure in dogs. (PDF)

S1 Table [^11^C]DASB administration parameters. (DOCX)
