# Supplementary material for: In Vivo Evaluation of Blood Based and Reference Tissue Based PET Quantifications of [11C]DASB in the Canine Brain
Source: PLoS One. 2016 Feb 9;11(2):e0148943. doi: 10.1371/journal.pone.0148943 (PMC4747581; doi:10.1371/journal.pone.0148943)
Supplement: S1 File — (PDF) [file pone.0148943.s001.pdf]

In vivo evaluation of blood based and reference tissue based PET quantifications of [^{11}C]DASB in the canine brain

Supporting data: Revalidation of the SPE-purification procedure in dogs

a) Beagle plasma spiked with [^{11}C]DASB.

➔ Three beagle plasma samples were spiked with a small amount of [^{11}C]DASB. SPE purification was done according to the protocol described by Ginovart and co-workers [1]. Each of the fractions, the THF fraction, the other fractions and the cartridge were counted for radioactivity using a calibrated 3x3 inch NaI(Tl) scintillation detector (Canberra, Meriden, Connecticut, USA). The ratio of the activity in the THF fraction to the total activity was determined to be ≥ 94%.

Sample	THF fraction (kBq)	Other fractions (kBq)	Leftovers on HLB cartridge (kBq)	THF-ratio
**1**	238	3	10	0.95
**2**	312	6	15	0.94
**3**	444	4	25	0.94

a) Blood sample of beagle taken 10 min after [^{11}C]DASB injection

➔ A plasma sample was taken 10 minutes after injection of [^{11}C]DASB in one of the beagles. After SPE purification, the THF fraction was evaporated to near dryness, taken up in 800 µL HPLC buffer, and analysed by HPLC for metabolites. HPLC analysis was performed using an Alltima C18 column (10 µm, 250x10 mm) and a 0.05 M ammonium acetate buffer pH 5.5/acetonitrile 70/30 (V/V) mobile phase at 8 mL/min with inline UV- and radioactivity detectors. The eluate was collected in fractions of 2 min and all fractions were counted for radioactivity using a calibrated 3x3 inch NaI(Tl) scintillation detector (Canberra, Meriden, Connecticut, USA). As the retention time of the DASB reference standard was 9.5 minutes (UV-detection) and a delay between the UV-detection and the collection of the samples of approximately 1 to 1.5 min can be expected, these results indicate that HPLC analysis of the THF fraction showed only the presence of unmetabolized [^{11}C]DASB.

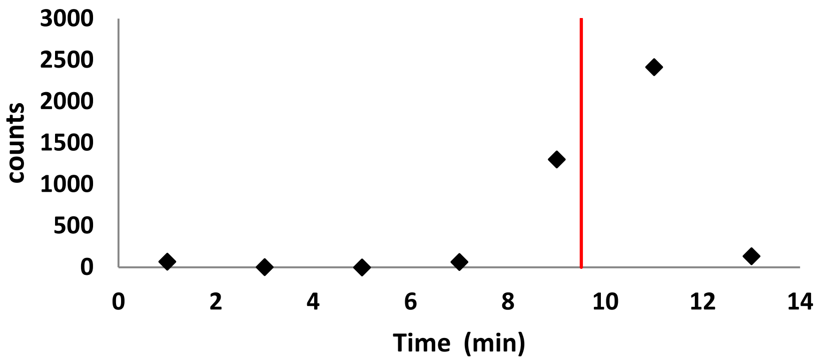

*red line = retention time of DASB reference standard (UV-detection)
