# Supplementary material for: In Vivo Evaluation of Blood Based and Reference Tissue Based PET Quantifications of [11C]DASB in the Canine Brain
Source: PLoS One. 2016 Feb 9;11(2):e0148943. doi: 10.1371/journal.pone.0148943 (PMC4747581; doi:10.1371/journal.pone.0148943)
Supplement: S1 Table — (DOCX) [file pone.0148943.s002.docx]

|  | Beagle 1 | Beagle 2 | Beagle 3 | Beagle 4 | Beagle 5 |
| --- | --- | --- | --- | --- | --- |
| Dog weight (kg) | 17 | 32 | 29 | 11 | 11 |
| Injected activity (MBq) | 194 | 269 | 316 | 347 | 319 |
| Injected mass (µg/kg) | 0,42 | 0,11 | 0,07 | 0,18 | 0,09 |
| SERT-occupancy (%) | 2,59 | 0,72 | 0,44 | 1,18 | 0,53 |
